# Performance Evaluation and Optimization of Dyes Removal using Rice Bran-Based Magnetic Composite Adsorbent

**DOI:** 10.3390/ma13122764

**Published:** 2020-06-18

**Authors:** Chih Ming Ma, Gui Bing Hong, Yi Kai Wang

**Affiliations:** 1 Department of Cosmetic Application and Management, St. Mary’s Junior College of Medicine, Nursing and Management, No. 100, Lane 265, San-Shing Rd., Sec. 2, San-Shing Shiang, YiLan 266, Taiwan; 2 Department of Chemical Engineering and Biotechnology, National Taipei University of Technology, No. 1, Sec. 3, Zhongxiao E. Rd., Taipei 10608, Taiwan; lukehong@ntut.edu.tw (G.B.H.); toro520191@gmail.com (Y.K.W.)

**Keywords:** rice bran, green composite, dye adsorption, sustainable development

## Abstract

Although several studies have explored green adsorbent synthesized from many types of agriculture waste, this study represents the first attempt to prepare an environmentally friendly rice bran/SnO_2_/Fe_3_O_4_-based absorbent with economic viability and material reusability, for the promotion of sustainable development. Here, rice bran/SnO_2_/Fe_3_O_4_ composites were successfully synthesized and applied for adsorption of reactive blue 4 (RB4) and crystal violet (CV) dyes in aqueous solutions. The adsorption data were well-fitted by the Langmuir isotherm model and the pseudo-second-order kinetic model. The maximum adsorption capacities of the RB4 and CV dyes as indicated by the Langmuir isotherm model were 218.82 and 159.24 mg/g, respectively. As results of response surface methodology (RSM) showed, the quadratic model was appropriate to predict the performance of RB4 dye removal. The findings exhibited that an optimum removal rate of 98% was achieved at 60 °C for pH 2.93 and adsorption time of 360 min. Comparative evaluation of different agricultural wastes indicated that the rice bran/SnO_2_/Fe_3_O_4_ composite appeared to be a highly promising material in terms of regeneration and reusability, and showed that the composite is a potential adsorbent for dye removal from aqueous solutions. Overall, the study results clearly suggest that an adsorbent synthesized from rice bran/SnO_2_/Fe_3_O_4_ magnetic particle composites provides encouraging adsorption capacity for practical applications for environmental prevention.

## 1. Introduction

Various environmental protection problems (e.g., water pollution) have resulted from the rapid development of industrialization and the significant increase in the human population. Dyes are widely used in many industries and can be harmful to aquatic life, the food web, and humans [1,2]. In fact, the presence of dyes in wastewater may reduce sunlight penetration, exhaust dissolved oxygen, prevent photosynthesis, and accumulate mutagenic and carcinogenic intermediates via chemical or biological reactions [3,4]. The effective removal of dyes from wastewater is one of the major environmental challenges in water management [5]. To date, various methods of wastewater treatment (e.g., biological treatments, chemical oxidation, coagulation/flocculation, ion exchange, membrane filtration, ozone treatment, and photocatalysis) have been used to remove dyes from aqueous solutions [6]. Among these treatment methods, the adsorption method is the most widely used because of its simple operational procedures, system flexibility, economic feasibility, and low energy consumption to produce high-quality treated effluent without secondary pollution [7,8]. To explore low-cost and environmentally friendly adsorbents, various agro-industrial wastes (e.g., walnut shells, mango stones, peanut husks, coir pith, sawdust, bamboo, wheat straw, rice bran, coal bottom ash, blast furnace slag, and orange peel) have been used as a result of their source abundance, recyclability, and economic viability [9,10,11,12,13,14,15]. However, most of the adsorbents synthesized from agriculture wastes have some limitation, such as lower regenerative capacity and adsorption capacity.

The unique characteristics of nanoparticles (NPs) (e.g., high dispersibility and a high surface-to-volume ratio) can provide significant adsorption capacity and are of considerable interest to the scientific community [16,17]. Recently, metal oxide nanocomposites (e.g., MnO_2_/MCM-41, Fe_2_O_3_/SiO_2_, CuS/ZnS) have been treated as efficient adsorbents for the removal of pollutants from aqueous solutions and have also exhibited the characteristics of good regeneration ability [12,17,18]. Moreover, as several studies revealed, magnetic nanoparticles (e.g., graphene-Fe_3_O_4_, NiFe_2_O_4_, and Fe_3_O_4_/C) are well-performing adsorbents which can be recovered quickly by an external magnetic field after application [19,20,21]. Although composite nanomaterials for dye removal have been reported, few studies have been undertaken on the various adsorption applications of environment-friendly nanocomposite adsorbent produced from agriculture byproducts or wastes. However, clear strategies for alternatives using the most promising composite materials for dye or pollutant removal remain to be uncovered.

Recently, response surface methodology (RSM) has been popularly applied to increase the efficiency of contaminant removal in environmental remediation [22,23]. RSM can quantitatively evaluate interactive effects of multiple variables and effectively reduce the number of experimental variables. RSM has been applied to various wastewater treatments, such as the Fenton process, electrocoagulation, photocatalysis, membranes, and adsorption operations for optimization [24,25,26,27,28]. In addition, regarding recycled agricultural materials, rice bran, also known as miller’s bran, is a common agricultural byproduct in Taiwan and can be obtained during the milling of rice. The major components of rice bran are vitamins, dietary fiber, essential fatty acids, minerals, and other sterols [29]. The functional groups in the rice bran (e.g., carboxyl and hydroxyl substituents) can considerably enhance the efficiency of dye for adsorption, making rice bran a potential adsorbent for removing dyes from aqueous solutions [12].

The purpose of this study was to synthesize a low-cost, reusable, and environmentally friendly adsorbent with highly promising capabilities of dye adsorption. Moreover, the RSM method was adopted to optimize removal efficiency and analyze the factor interactions for maximal performance. This study specifically synthesized a rice bran/SnO_2_/Fe_3_O_4_ composite absorbent for adsorption of reactive blue 4 (RB4) and crystal violet (CV) dyes in aqueous solutions. For system optimization, central composite design (CCD) was selected to globally optimize the three control factors of RB4 dye performance. To determine statistical significance, analysis of variance (ANOVA) was used to evaluate the data validation of the RSM model. To reveal detailed characterization, the rice bran-based composite adsorbents were characterized via scanning electron microscopy (SEM), energy-dispersive X-ray (EDX), Fourier transform infrared (FTIR) spectroscopy, and Brunauer–Emmett–Teller (BET) specific surface area analysis techniques. Moreover, the adsorption behavior of the dyes was analyzed using reaction kinetics and isotherm models. For sustainable uses, the regeneration performance of the composite adsorbent was also investigated in this study. To the best of our knowledge, this work is the first attempt to prepare an environmentally friendly rice bran/SnO_2_/Fe_3_O_4_ adsorbent.

## 2. Materials and Methods

### 2.1. Materials

Methanol (purity ≥ 99.9%), ethanol (purity ≥ 99.8%), tin (II) chloride dehydrate, and sodium hydroxide (purity ≥ 97%) were purchased from Fisher Chemical (Pittsburgh, PA, USA). Sodium carbonate (purity ≥ 99%), ferric chloride (purity ≥ 99%), ferrous chloride (purity ≥ 99%) and crystal violet (CV) were obtained from Acros Organics (Geel, Belgium). Reactive blue 4 (RB4) was supplied by Sigma-Aldrich (Natick, MA, USA). All chemicals were analytical grade and used as received. Rice bran was harvested from a local Taichung grinding mill in Taiwan. After cleaning, the rice bran was dried for 6 h in an oven at 110 °C. The dried rice bran was ground in a mill and sieved (20 and 100 mesh) and fractions of 0.15–0.85 mm were selected for the adsorbent preparation.

### 2.2. Synthesis of Rice Bran/SnO_2_/Fe_3_O_4_ Composite

**Figure 1:**
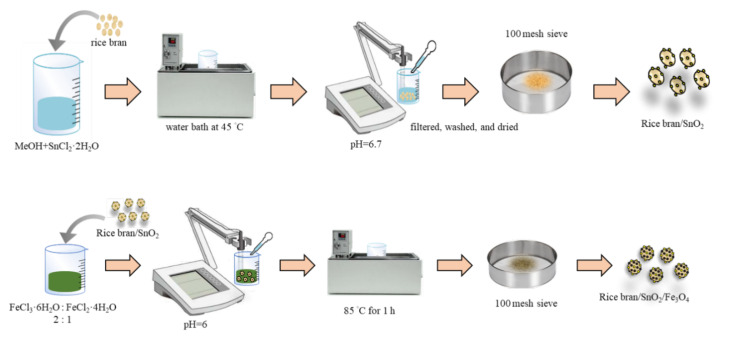
Preparation schematic diagram of rice bran/SnO_2_/Fe_3_O_4_ composite.

As indicated in previous research [12], rice bran has been successful modified to be an appropriate adsorbent. The present study modified this adsorbent to a rice bran/SnO_2_/Fe_3_O_4_ composite to significantly augment adsorption efficiency. The modified method of preparing composite materials is shown in Figure 1. After mixing 15 mL of the precursor (0.1 M SnCl_2_·2H_2_O) and 35 mL of methanol, 2 g of the rice bran was added to the mixed solution. Subsequently, the mixed solution was then kept in a water bath at 45 °C for 40 min. Then, the pH of the solution was adjusted to 6.7 and the solution was kept in an isothermal water bath for 15 min. The obtained product was filtered and washed several times with deionized water, and then dried in an oven at 50 °C for 6 h. The dried precipitate was ground and then sieved (100 mesh) to obtain the rice bran/SnO_2_ composite. Then, the magnetite rice bran/SnO_2_ composite (rice bran/SnO_2_/Fe_3_O_4_) was synthesized by the chemical co-precipitation method [30]. Briefly, rice bran/SnO_2_ (2 g), ferric chloride (2.7 g), and ferrous chloride (1.2 g) were dissolved in deionized water (100 mL). Sodium carbonate (2.5 M) was slowly added to the mixture to adjust the pH level to 6.0. Then, the mixture was placed in a shaker at 85 °C for 1 h at a constant speed of 100 rpm. After serial procedures of filtering, washing, and drying (50 °C for 6 h), the rice bran/SnO_2_/Fe_3_O_4_ composite was obtained.

### 2.3. Composite Adsorbent Characterizations

Surface morphology of adsorbents was observed by a scanning electron microscope (S-3000H, Hitachi Science & Technology, Tokyo, Japan). The element identification and distribution of rice bran/SnO_2_/Fe_3_O_4_ composite was analyzed by EDX (Emaxx-act, Horiba, Kyoto, Japan). The surface functional groups on adsorbents were investigated using FTIR Spectroscopy (FT/IR-6600 Series, Jasco, Tokyo, Japan) coupled with single reflection ATR (ATR Pro One) within the wavelength range of 4000–400 cm^−1^. Brunauer–Emmett–Teller (BET, Micromeritics, Norcross, GA, USA) measurements were made using a Micromeritics’ Gemini™ V Series 2380 analyzer. The specific surface area and pore area distribution of the adsorbent were specifically analyzed. The zero-point charge (pHzpc) of rice bran/SnO_2_/Fe_3_O_4_ composite adsorbent was determined using the salt addition method.

### 2.4. Batch Adsorption Experiments

The batch dye adsorption experiments were carried out in Erlenmeyer flasks in a temperature-controlled water bath shaker at 100 rpm. The effects of control variables, such as the pH (2–10) of the dye solution, temperature (30–60 °C), adsorbent dose (0.5–3.5 g/L), and contact time (0–360 min) were studied. As indicated by the literature, for comparison the dye concentrations in water streams were approximately 10–200 mg/L [31]. Therefore, the adsorption experiment was executed at a fixed dye initial concentration of 200 mg/L. One control factor was varied to investigate the individual effect of each control factor on the adsorption performance of the adsorbent, while all other control factors were maintained constant. The contents of the CV and RB4 dyes adsorbed onto the adsorbent were determined using a UV/VIS spectrophotometer (Thermo Scientific, Waltham, MA, USA) at maximum wavelengths of 590 and 595 nm, respectively. All the experiments were carried out in triplicate to confirm data reproducibility of the results. The percentage dye removal was calculated according to the following equation and used in the ensuing adsorption experiments:

(1) $$\mathrm{Dye}\;\mathrm{removal}\;\left(\%\right)=\frac{C_{0}-C_{e}}{C_{0}}\times 100\%$$

(2) $$\mathrm{Adsorption}\;\mathrm{capacity}\;\left(q_{e}\right)=\frac{C_{0}-C_{e}}{W}\times V$$

where *C*_0_ and *C_e_* are the initial and equilibrium dye concentrations (mg/L), respectively; *q_e_* (mg/g) is the adsorption capacity at equilibrium; and V (L) and W (g) are the volume of the dye solution and the dosage of adsorbent used, respectively.

### 2.5. Experimental Optimization of Adsorption

To show the full impacts of adsorption, RSM can be used to maximize or minimize the output response and analyze the factor interactions [32]. The adsorption performance was optimized by CCD based on RSM. According to preliminary results of dye adsorption, contact times, temperature, and pH might each affect adsorption performance. Therefore, the variables of contact time, temperature, and pH were chosen for the experimental design of RSM in experimental optimization, acquiring the optimum adsorption conditions for the removal efficiency of RB4. In the optimization work, 20 experiments (8 level points, 6 axial points, and 6 replicates at the center points) were conducted by experimental design, as shown in Table 1. In this study, ANOVA was used to check and evaluate the validation of the RB4 removal quadratic model. To investigate the interactions between dependent variables, 3D surface plots and ANOVA results were also derived.

## 3. Results and Discussion

### 3.1. Characterization of Adsorbent

**Figure 2:**
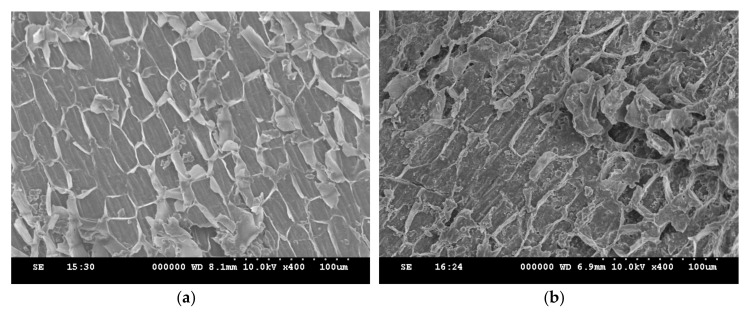

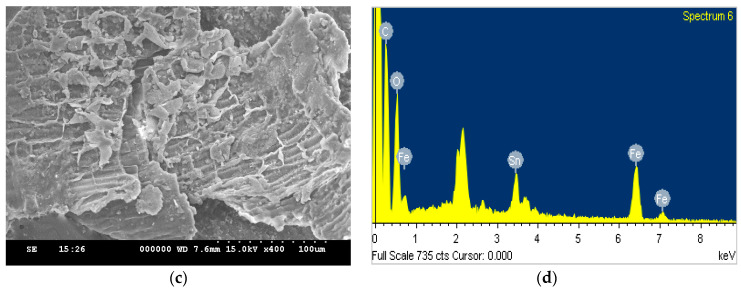
Scanning electron microscopy (SEM) images of the adsorbents: (**a**) rice bran, (**b**) rice bran/SnO_2_, and (**c**) rice bran/SnO_2_/Fe_3_O_4_; and (**d**) energy-dispersive X-ray (EDX) spectrum of rice bran/SnO_2_/Fe_3_O_4_.

As shown in Figure 2, the micrograph of the rice bran (Figure 2a) exhibited a fiber cell structure of a hexagonal or pentagonal shape with a smooth surface and without pores. After SnO_2_ was loaded on the rice bran to obtain the rice bran/SnO_2_ composite, the surface was clearly damaged (Figure 2b). With the magnetizing process, the surface of the rice bran/SnO_2_/Fe_3_O_4_ (Figure 2c) composite was apparently more seriously damaged, allowing magnetic particles to be present. Moreover, EDX analysis of the rice bran/SnO_2_/Fe_3_O_4_ clearly confirmed the presence of atomic species of C, O, Fe, and Sn (Figure 2d).

**Figure 3:**
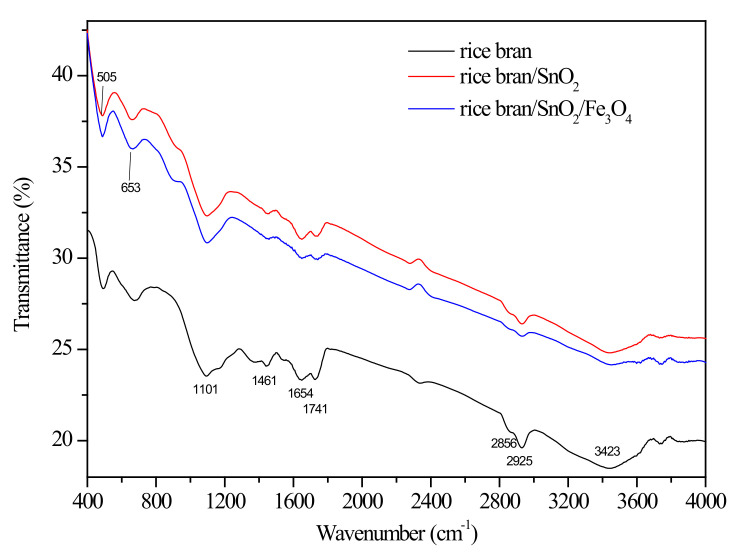
FTIR spectra of the rice bran, rice bran/SnO_2_, and rice bran/SnO_2_/Fe_3_O_4_.

Figure 3 shows the comparison of FTIR spectra of the rice bran, rice bran/SnO_2,_ and rice bran/SnO_2_/Fe_3_O_4_. The presence of rice bran evidently provided a strong and broad absorption peak vibration (-OH and -NH_2_) at ca. 3600–3200 cm^−1^, possibly combining the contributions from gamma-oryzanol, ferulic acid, cellulose, and lignin [33,34]. The peaks in the rice bran spectrum at 2925 cm^−1^ and 2856 cm^−1^ are assigned to the -CH stretching vibrations, which are likely due to the contributions of cellulose and hemicellulose [35]. Other characteristic peaks at 1741, 1654, 1640 and 1200–1100 cm^−1^ represent the C=O, C=C, -NH and C-O groups, respectively [36]. A new peak in the FTIR spectrum appeared at 505 cm^−1^ of rice bran/SnO_2_, which is the stretching vibration of Sn-O [37]. After the magnetization, another new peak appeared at 653 cm^−1^ of rice bran/SnO_2_/Fe_3_O_4_, which is the stretching vibration of Fe-O [4]. This also indicated that the SnO_2_ and Fe_2_O_3_ were indeed attached to the rice bran. After the SnO_2_ and magnetic particles were loaded into the surface of the rice bran, the transmittance intensity was significantly attenuated.

**Figure 4:**
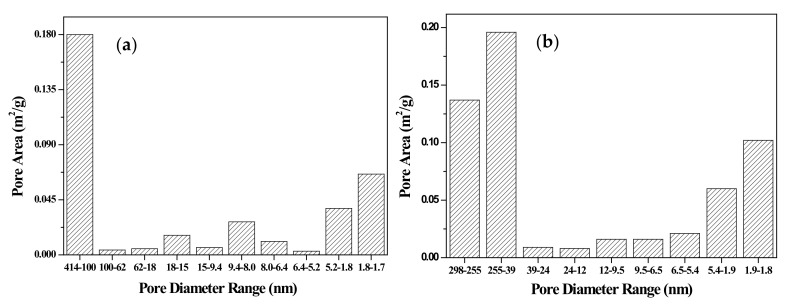

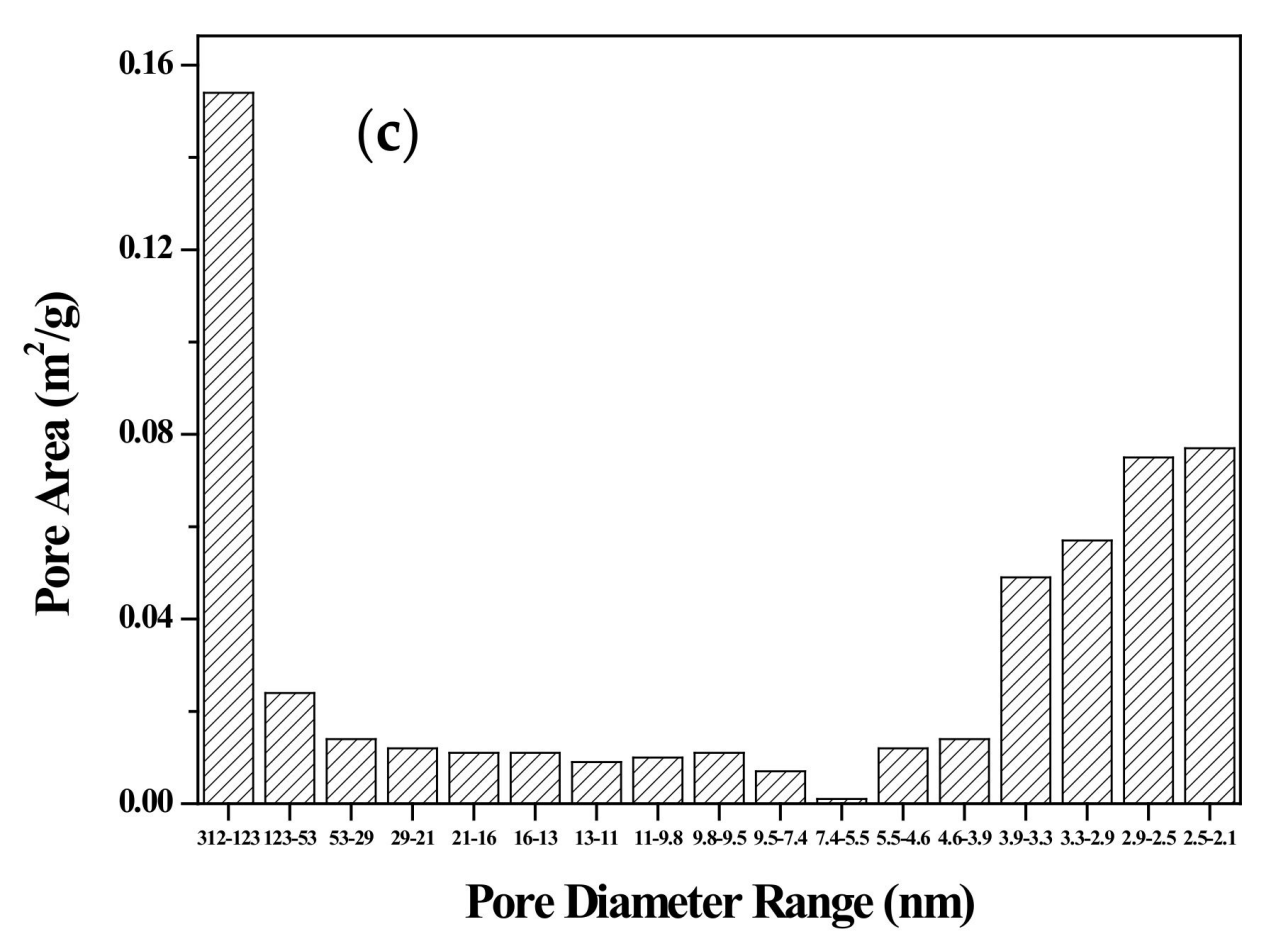
Pore area distribution of different pore size ranges on the adsorbent: (**a**) rice bran, (**b**) rice bran/SnO_2_, and (**c**) rice bran/SnO_2_/Fe_3_O_4_.

The specific surface area and porosity of the adsorbent are also characteristics of significant importance as indicators to characterize the adsorption behavior. Figure 4 shows the pore area distribution of the different pore sizes in the adsorbent. Most pore areas in the rice bran and rice bran/SnO_2_ (Figure 4a,b) are macropores (>50 nm). In addition, the pore areas of mesopores (2–50 nm) and micropores (<2 nm) in SnO_2_ are clearly higher than those in rice bran. For the rice bran/SnO_2_/Fe_3_O_4_ composite (Figure 4c), the pore area increased, particularly in the pore size range of 2–4 nm, which was likely due to the presence of SnO_2_ and magnetic particles. To provide a detailed figure of the available adsorption sites on the composite surface, BET analysis of the adsorbents was implemented, as indicated in Table 2. The BET specific surface area of the composites increased due to the presence of SnO_2_ and magnetic particles. The rice bran/SnO_2_/Fe_3_O_4_ composite had the largest specific surface area (0.746 m^2^/g) because of the attachment of SnO_2_/magnetic particles onto the surface of the rice bran. Thus, due to such a significant increase in contact area, rice bran/SnO_2_/Fe_3_O_4_ was anticipated to adsorb more dyes onto the surface of the adsorbent.

### 3.2. Effect of the Operation Condition

#### 3.2.1. Effect of pH

To reveal figures of absorption and desorption of prepared composites, the solution pH value is one of the most important influences on the adsorption process. The pH level affects the surface charge density between the adsorbent and the adsorbate to influence the adsorption capacity of the adsorbent. As Chakraborty et al. (2011) indicated, the surface of the adsorbent tends to be positively charged under acidic conditions, leading to a decrease in the available sites of adsorption of positively charged cationic dye due to electrostatic repulsion [10]. Munagapati and Kim (2016) reported that the number of negatively charged sites increases with the increase in the pH of the solution, and the surface of the adsorbent tends to be negatively charged under alkaline conditions. In contrast, adsorption of anion dye is not effective under higher pH conditions [11].

**Figure 5:**
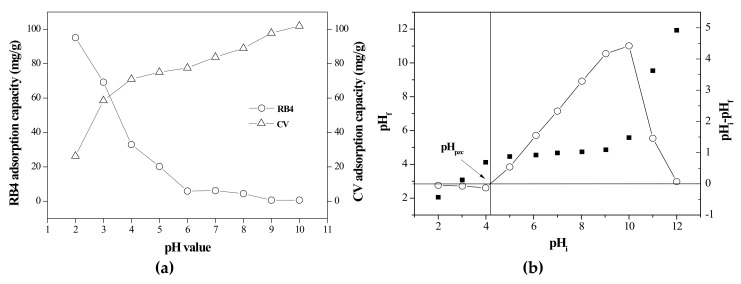
(**a**) pH effect on dye removal in rice bran/SnO_2_/Fe_3_O_4_ and (**b**) the determination of pH_pzc_ of rice bran/SnO_2_/Fe_3_O_4_.

Figure 5a shows the effect of pH on the adsorption capacities of RB4 and CV onto the rice bran/SnO_2_/Fe_3_O_4_ adsorbent (2 g/L) at 30 °C for 360 min. The adsorption of RB4 decreased and that of CV rose as the pH of the aqueous dye solution increased. This could be due to on pH_PZC_ of the adsorbent. At a low pH (<5.0), the surface of rice bran/SnO_2_/Fe_3_O_4_ is positively charged (pH_PZC_ = 4.18, as shown in Figure 5b), which is due to the protonation of functional groups such as amines, carboxyls, and hydroxyls on the adsorbent. As a result, the interaction between the anion dye (RB4) and the adsorbent was favorable and the adsorption of RB4 increased. At a high pH (>5.0), the surface of the adsorbent was negatively charged. Moreover, the adsorption of the cationic dye (CV) increased with increasing pH due to the enhancement of electrostatic attraction that resulted from the increase in the negatively charged surface sites on the adsorbent [38]. This result of the pH effect was similar to those of different adsorbents based on agricultural resources as indicated elsewhere [11,12]. However, precipitation of CV was observed at pH = 10. Therefore, additional dye studies for RB4 and CV were conducted at fixed pH values of 2 and 9, respectively.

#### 3.2.2. Effect of Adsorbent Dose

**Figure 6:**
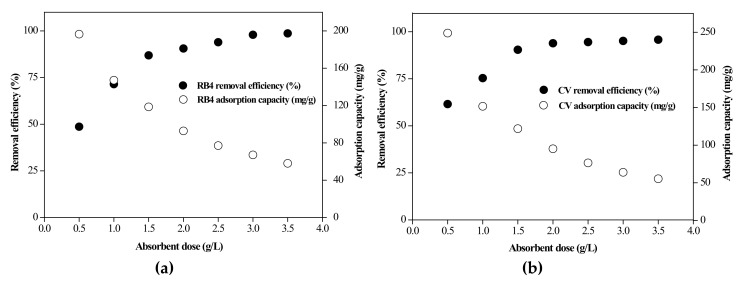
Effect of adsorbent dosage on the removal of (**a**) RB4 and (**b**) crystal violet (CV).

The dose of the adsorbent is also an important factor for dye adsorption, as it can determine the capability of dye removal and affect the cost of the dye solution. The effect of the adsorbent dose on the removal of the RB4 and CV dyes was evaluated over a range of 0.5–3.5 g/L. Figure 6 shows the effect of the adsorbent dose on the dye removal efficiency and the dye adsorption capacity of RB4 and CV onto the rice bran/SnO_2_/Fe_3_O_4_ composite. Both the RB4 and CV dye removal efficiencies were positively correlated with the adsorbent dose and gradually approached equilibrium at an adsorbent dose of 1.5 g/L. In contrast, the adsorption capacity was negatively correlated with the composite dose. The decrease in the adsorption capacity at a higher adsorbent dose may be due to aggregation [39,40]. Such aggregation decreases the surface area and available active sites of the composite and increases the diffusional path length [41]. At an adsorbent dose of 1.5 g/L, the dye removal efficiency reached approximately 90%, and dye removal was not significantly affected as the adsorbent dose was further increased. Therefore, the optimum dose for effective removal of RB4 and CV dyes was found to be 1.5 g/L.

#### 3.2.3. Effect of Temperature

**Figure 7:**
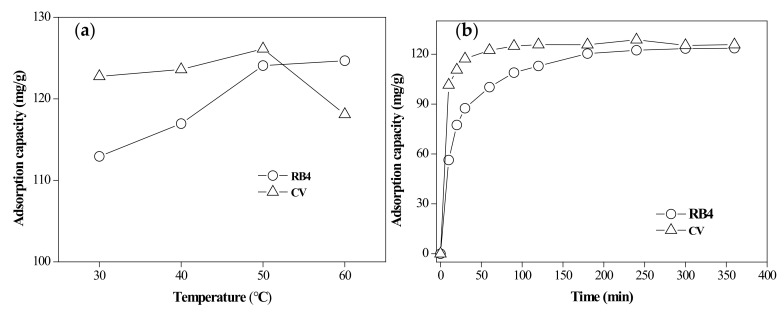
(**a**) Effect of temperature on removing dyes; (**b**) effect of contact time on removing dyes.

The influence of temperature on the adsorption capacity of the dyes was investigated in the range of 30–60 °C (Figure 7a). The dyes’ adsorption capacities for the adsorbent increased as the temperature increased from 30 to 50 °C. At a higher temperature, the viscosity of the solution decreased, which helped the dye molecules to rapidly penetrate the outer boundary layer and diffuse into the pores of the adsorbent. On the other hand, the increase in temperature caused the expansion of the pores and increased the contact ability between the adsorbate and the adsorbent to facilitate adsorption of the dyes. RB4 adsorption at 60 °C was not significantly changed, suggesting the stability of the composite to hold dye molecules. However, the CV adsorption at 60 °C decreased, possibly due to the damage of the active sites, which weakened the force between the active binding sites of the adsorbent and the adsorbate molecules [42,43]. Hence, the optimum adsorption temperature for dyes was found to be 50 °C in this study.

### 3.3. Adsorption Kinetics and Isotherm Modeling

#### 3.3.1. Adsorption Kinetics

An investigation of the dye adsorption kinetics was carried out using the single-factor optimal conditions (RB4 dye initial concentration of 200 mg/L, temperature 60 °C, pH = 2, and adsorbent dose of 1.5 g/L; CV dye initial concentration of 200 mg/L, temperature 50 °C, pH = 9, and adsorbent dose of 1.5 g/L). Figure 7b shows the adsorption capacity for RB4 and CV for various contact times (<6 h). Evidently, the adsorption capacity increased with contact time and reached equilibrium after 6 h. The adsorption kinetics of RB4 and CV onto the rice bran/SnO_2_/Fe_3_O_4_ composite tended to be described by the pseudo-first-order model (PFOM) and pseudo-second-order model (PSOM), which can be presented as Equations (3) and (4), respectively [44,45]:

(3) $$\ln\left(q_{e}-q_{t}\right)=\ln q_{e}-k_{1}t$$

(4) $$\frac{t}{q_{t}}=\frac{1}{k_{2}q_{e}^{2}}+\frac{t}{q_{e}}$$

where *k*_1_ (min^−1^) and *k*_2_ (g/mg·min) are the adsorption rate constant of PFOM and PSOM, respectively; *q_t_* (mg/g) is the adsorption capacity at time t (min).

The results in Table 2 show that the experimental *q_e_* values seemed to be more consistent with the calculated values of the PSOM, and the linear regression coefficient (*R*^2^) is the highest among these models. Therefore, the PSOM is more suitable than the PFOM to describe the transient dynamics of the adsorbent adsorption.

In fact, the dye adsorption process on composite adsorbent was complicated, and possibly different mechanisms were involved. An intraparticle diffusion model (IPDM) can be applied to describe the diffusion mechanism of the dye molecules from the surface to bulk of the adsorbent as described elsewhere [46]:

(5) $$q_{t}=k_{id}t^{0.5}+C$$

where *k_id_* (mg/g·min^0.5^) is the intraparticle diffusion rate constant; *C* is the boundary layer thickness.

The estimated parameters of the IPDM are listed in Table 3. The kinetic data might be composed of multiple linearities and two or more stages that may influence dye adsorption, and it appeared intraparticle diffusion was not the only rate-controlling step. The first stage is instantaneous adsorption (external mass transfer or boundary layer effect) when dye molecules are adsorbed onto the adsorption sites available on the surface of the adsorbent. The second stage (pore diffusion) consists of dye molecules diffusing into the bulk of the adsorbent and finally approaching the equilibrium stage. A larger intercept, *C* (boundary layer thickness), led to a greater boundary layer effect on the adsorption process [36]. In addition, a smaller boundary layer thickness (*C*) and a larger intraparticle diffusion rate constant (*k_id_*) indicated that the dye adsorption process was mostly governed by boundary layer diffusion [8].

#### 3.3.2. Isotherm Modeling

The adsorption isotherm model can be described as the distribution of dye molecules between the liquid (dye solution) and solid (adsorbent) phases in an equilibrium state. Adsorption isotherm experiments were carried out at a constant temperature with different initial concentrations (200, 300, 400, 500, and 600 mg/L). To study the most suitable model for the adsorption of RB4 and CV dyes, the widely used Langmuir and Freundlich isotherm models were used to fit the adsorption isotherm data; these models can be expressed as Equations (6) and (7), respectively [47,48]:

(6) $$\frac{C_{e}}{q_{e}}=\frac{1}{q_{m}K_{L}}+\frac{C_{e}}{q_{m}}$$

(7) $$\ln q_{e}=\frac{1}{n}\ln C_{e}+\ln K_{F}$$

where *C_e_* is the dye concentration (mg/L) at equilibrium; *q_m_* and *K_L_* are the maximum adsorption capacity (L/mg) and the Langmuir constant, respectively; *K_F_* and *n* are the Freundlich isotherm constant (mg/g) and adsorption intensity, respectively.

**Figure 8:**
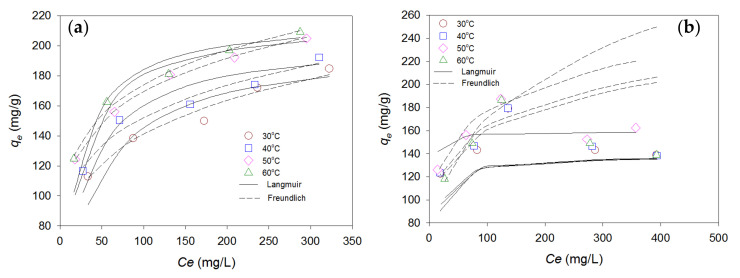
Adsorption capacity at various temperature of dye adsorption onto rice bran/SnO_2_/Fe_3_O_4_ composite adsorbent: (**a**) RB4; (**b**) CV.

Figure 8 shows the equilibrium adsorption of RB4 and CV onto rice bran/SnO_2_/Fe_3_O_4_ composite adsorbent (pH = 2, adsorbent dosage of 1.5 g/L, and RB4 concentration of 200 mg/L; pH = 9, adsorbent dosage of 1.5 g/L, and CV concentration of 200 mg/L), respectively. The adsorption capacities increase at higher temperature of RB4 dye, which was consistent with previous experimental results of the temperature effect. The analysis results of the isotherm adsorption data are shown in Table 4. The isotherm adsorption data (*q_e_* and *C_e_*) was fitted to Langmuir model and Freundlich model, as shown in Figure 8. The linear regression coefficient (*R*^2^) of the Langmuir isotherm model for both dyes was greater than the *R*^2^ value of the Freundlich model, indicating that the Langmuir isotherm model was more appropriate to describe the isotherm adsorption behavior than the Freundlich model. The Langmuir adsorption model elucidates adsorption by assuming monolayer coverage, insignificant interactions between adsorbate molecules on adjacent sites, and energetically identical sites for adsorption. Therefore, this finding may suggest that the RB4 and CV dyes were adsorbed onto a homogeneous surface of the adsorbent and formed a single layer with uniform adsorption energies. The maximum adsorption capacities of the RB4 and CV dyes were 218.82 and 159.24 mg/g, respectively. As shown in the literature, RB4 and CV dyes have been removed by adsorbents produced from different agriculture wastes [49,50,51,52,53,54]. The adsorption capacities of the composite adsorbent developed in this study compared with those of various agricultural waste adsorbents, for removal of RB4 or CV from aqueous solutions, were found to be highly promising (Table 5). The results suggest that the newly developed rice bran/SnO_2_/Fe_3_O_4_ composite adsorbent can provide better adsorption capacity than other adsorbents. The rice bran/SnO_2_/Fe_3_O_4_ composite adsorbent also has better adsorbing capacity than rice bran/Fe_3_O_4_ [12]. This new composite adsorbent can be more favorably used in removal of both cationic and anionic dyes.

### 3.4. Optimal Adsorption and Interactive

To determine global optimization of adsorption, optimization of the main factors and two-factor interaction should be assessed. Thus, the results of RB4 removal rate based on the CCD of the RSM are shown in Table 1. The experimental data were applied to evaluate linear, quadratic, and cubic models, and results indicated that the quadratic model was highly significant and suitable to represent the response of the RB4 dye removal. Assuming the combined interaction of more than two factors could be neglected, a second-order polynomial equation was obtained, and the calculation result of the RB4 removal rate equation was as follows:

(8) $$\mathrm{RB}4\;\mathrm{removal}\;\mathrm{rate}=31.41+7.06\;\times \;A+5.68\;\times \;B-24.09\;\times \;C-0.41\;\times \;\mathrm{AB}-\;3.97\;\times \;\mathrm{AC}-3.49\;\times \;\mathrm{BC}-2.32{\;\times \;A}^{2}+2.331\;\times \;10^{-5}{\times B}^{2}+4.51{\;\times \;C}^{2}$$

where A, B, and C are contact times, temperature, and values of pH, respectively. The predicted RB4 removal rate was computed by Equation (8); results are listed in Table 1.

The ANOVA results of the quadratic model are summarized in Table 6. The F-value, *p*-value, and lack of fit *p*-value of this empirical model were 98.84, less than 0.0001, and 0.1089, respectively. The results indicated that the empirical model is statistically significant with 95% confidence level. The determination coefficient (*R*^2^) was defined as a quality of the fitted model [55]. In this study, the *R^2^* was 0.9889, which showed that the predicted RB4 removal rate matches the experimental results. The predicted *R*^2^ is a measure of the level at which the fitted model predicts a response value. The acceptable range of the discrepancy between the adjusted *R*^2^ value and the predicted *R*^2^ value should be less than 0.20; if the numerical difference is above 2, the model is regarded as not being appropriate to fit the data [56]. In addition, the appropriate value of adequate precision should be higher than 4 [28]. In our work, the adjusted *R*^2^, predicted *R*^2^, and adequate precision were 0.9789, 0.9331, and 34.747 respectively.

The contact times (A), temperature (B), and values of pH (C) *p*-values were all are less than 0.05, which implied statistically significance, and indicated that the three control factors had direct effects on the RB4 removal rate. The weights of variables of A, B, and C in Equation (8) indicated that the significance effects on RB4 removal rate were ordered as follows: pH level > contact times > temperature. Furthermore, the higher F-value (as shown in Table 6) implied a larger influence and statistical significance in RB4 removal, which was unanimous with the weight of variables shown in the empirical model. Additionally, the *p*-values of AC, BC, A^2^, and C^2^ were all less than 0.05, and the data were found to be statistically significant for this quadratic model. According to Equation (8), both AC (contact time–pH value) and BC (temperature–pH value) had a negative influence on the RB4 adsorption efficiency.

**Figure 9:**
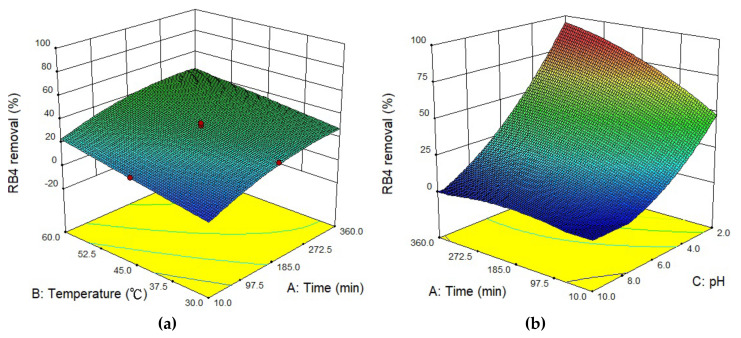

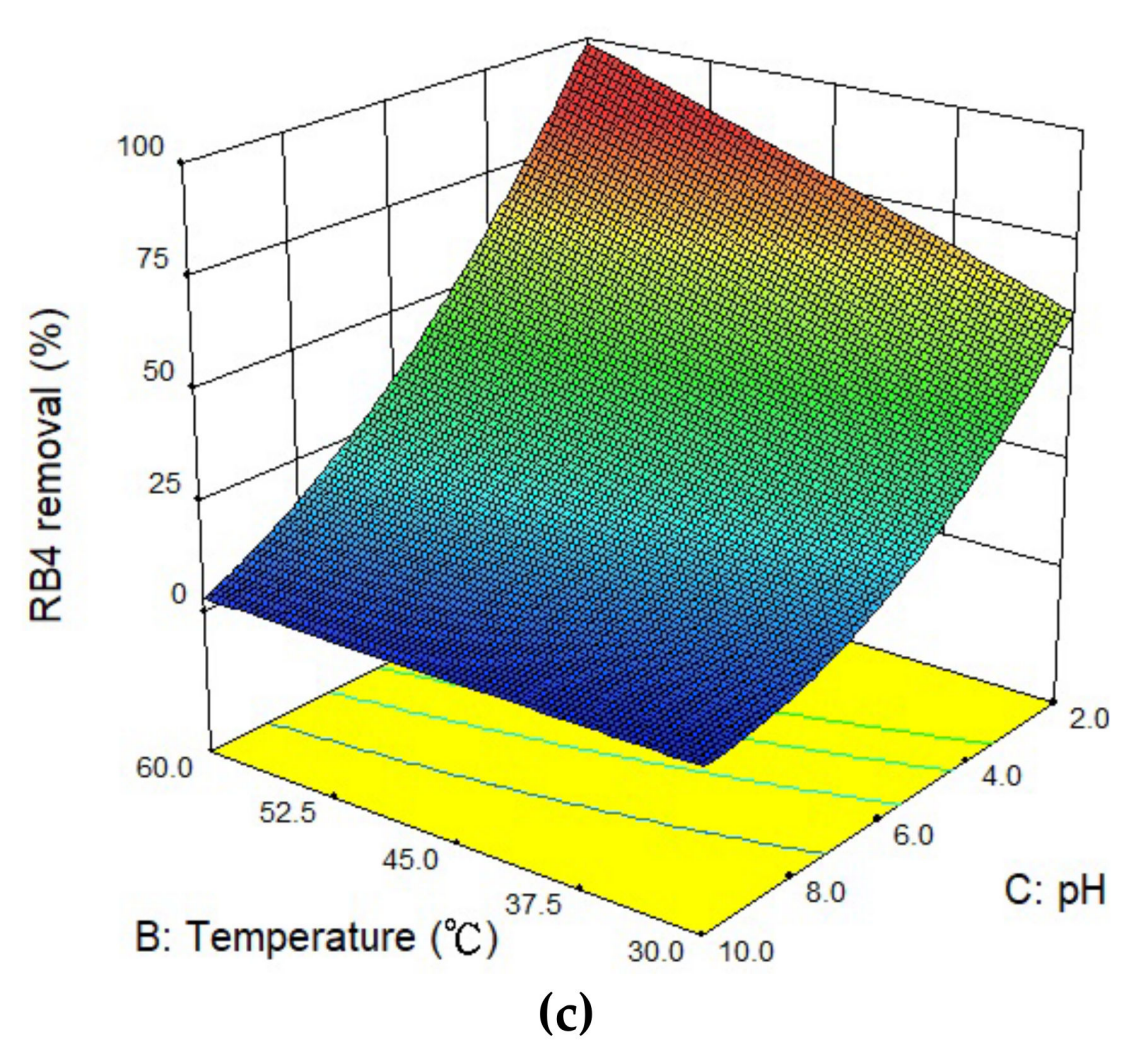
The 3D response surface plots of the removal rate of RB4 predicted by the response surface methodology (RSM) for (**a**) contact time and temperature, (**b**) contact time and pH, (**c**) pH and temperature.

In addition, 3D response surface plots were used to investigate the interactions among contact times, temperature, and pH values. As shown in Figure 9a, the 3D response surface was almost flat. Furthermore, according to the statistical results in Table 5, the interaction between contact time and temperature was also not statistically significant. It can be concluded that, although increased contact time and temperature both affected the removal rate of RB4, there was not a significant interaction between the contact time and temperature. From Figure 9b, it can be observed that increased contact time did not significantly impact the removal rate in a high pH solution. At pH = 2, it is clearly shown that the removal rate of RB4 increased significantly as the contact time increased. Figure 9c shows the relationship between pH value of the solution and reaction temperature. The 3D plot is a distorted plane, which indicates that the pH value of the solution has a significant interaction with temperature. The results demonstrate that this RSM empirical model is a feasible approach to analyze the factor interactions of RB4 dye removal.

To confirm the feasibility of the RSM model to assess the conditions of the optimal RB4 removal rate (temperature 60 °C, pH = 2.93, and adsorption time = 360 min), comparisons of predicted values and new experimental results under the optimal operating conditions were made. The prediction result of the optimal RB4 removal rate was 100%. The RB4 adsorption finding revealed a removal rate of 98% under optimal operating conditions. The result indicated an insignificant experimental error between predicted and experimental values of the optimum removal rate. Thus, the RSM optimization implemented was reliable and achieved maximum dye adsorption removal.

### 3.5. Regeneration of the Adsorbent

**Figure 10:**
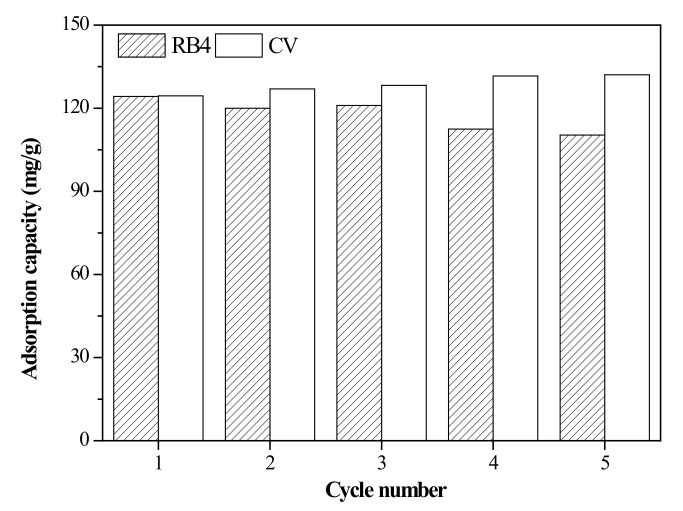
Regeneration performance of rice bran/SnO_2_/Fe_3_O_4_.

For green sustainability of the use of agricultural resources, the recycling and regenerative capacity of the adsorbent, as a crucial component of the cost-effectiveness of the water treatment process, was assessed. To investigate the reusability of the adsorbent, an acetic acid solution and a sodium hydroxide solution were used to desorb the RB4 and CV dyes. A dye-loaded adsorbent was immersed in a 0.1 M acetic acid solution and shaken for 10 min. The adsorbent was washed several times and immersed in a 0.1 M sodium hydroxide solution. Then, shaking was conducted for 10 min, and the adsorbent was then washed and dried to obtain the regenerated adsorbent. The regeneration performance of the adsorbent is shown in Figure 10. The adsorption capacity of RB4 decreased by 10% after five cycles, suggesting that the adsorbent has a very promising regeneration ability and reusability. In contrast, the adsorption capacity of CV increased by approximately 3% under the same conditions. That is, the adsorption capacity of CV was enhanced after the desorption process and was modified by the sodium hydroxide solution due to the surface characteristics of the rice bran/SnO_2_/Fe_3_O_4_ composite [57]. These results indicated that the rice bran/SnO_2_/Fe_3_O_4_ composite has a strong regeneration performance and can be reused as an adsorbent to efficiently remove RB4 and CV dyes at least five times.

## 4. Conclusions

The novelty of this work was the successful synthesis of a low-cost rice bran/SnO_2_/Fe_3_O_4_ composite and its application to the high capacity removal of RB4 and CV dyes from an aqueous solution. The adsorption performance of rice bran was enhanced by the loading of SnO_2_ and magnetic particles. In addition, compared to alternatives, the rice bran/SnO_2_/Fe_3_O_4_ composite possessed the largest specific surface area and was anticipated to adsorb more dyes onto its surface. The Langmuir isotherm model demonstrated a better fit to the isotherm adsorption, mono-layer adsorption seemed to appropriate. The results of the RSM showed that the quadratic model was suitable to represent the response of the RB4 dye removal. An optimum removal rate of 98% at 60 °C for pH = 2.93 and adsorption time = 360 min was found. Thus, the power of the model’s prediction of the optimum removal rate of RB4 dye appears to be highly promising for practical uses. In addition, the rice bran/SnO_2_/Fe_3_O_4_ composite showed excellent regeneration and reuse abilities. In the development and applicability of nanocomposite adsorbents, low-cost composites formed from agricultural byproducts as raw materials are economically feasible. The composite adsorbent developed in this study has the advantages of being recyclable and reusable, and has a simple preparation method, thus demonstrating its potential for the removal of dye from industrial wastewater.

## Figures and Tables

**Table 1:** Central composite design matrix and reactive blue 4 (RB4) dye removal.

Run	Independent Variables			Responses (RB4 Removal) (%)		Remark
	A: Contact Times	B: Temperature	C: pH	Predicted	Experimental	
1	185 (0)	60 (1.68)	6 (0)	40.95	36.36	axial point
2	360 (1.68)	45 (0)	6 (0)	36.72	33.58	axial point
3	81 (−1)	54 (1)	3.6 (−1)	56.24	56.33	level point
4	185 (0)	30 (−1.68)	6 (0)	21.87	23.66	axial point
5	290 (1)	54 (1)	3.6 (−1)	77.48	80.28	level point
6	185 (0)	45 (0)	10 (1.68)	3.68	0.13	axial point
7	185 (0)	45 (0)	2 (−1.68)	84.61	85.41	axial point
8	185 (0)	45 (0)	6 (0)	31.41	30.21	center point
9	185 (0)	45 (0)	6 (0)	31.41	35.32	center point
10	290 (1)	54 (1)	8.4 (1)	14.38	17.7	level point
11	185 (0)	45 (0)	6 (0)	31.41	30.01	center point
12	81 (−1)	36 (−1)	8.4 (1)	3.82	3.00	level point
13	290 (1)	36 (−1)	3.6 (−1)	59.96	58.82	level point
14	10 (−1.68)	45 (0)	6 (0)	13.00	13.33	axial point
15	81 (−1)	54 (1)	8.4 (1)	9.02	12.14	level point
16	185 (0)	45 (0)	6 (0)	31.41	32.79	center point
17	81 (−1)	36 (−1)	3.6 (−1)	37.08	35.75	level point
18	185 (0)	45 (0)	6 (0)	31.41	31.4	center point
19	290 (1)	36 (−1)	8.4 (1)	10.82	12.73	level point
20	185 (0)	45 (0)	6 (0)	31.41	29.21	center point

**Table 2:** Brunauer–Emmett–Teller (BET) surface area of the adsorbents.

Sample	BET Surface Area (m^2^/g)	Average Pore Volume (cm^3^/g)
rice bran	0.588	0.0058
rice bran/SnO_2_	0.699	0.0028
rice bran/SnO_2_/Fe_3_O_4_	0.746	0.0028

**Table 3:** Kinetic parameters for the adsorption of dyes onto rice bran/SnO_2_/Fe_3_O_4_ at 50 °C.

Dye	RB4	CV
*q_exp_* (mg/g)	123.58	125.71
PFOM		
*k*_1_ (1/min)	0.0089	18.8
*q_e_* (mg/g)	59.1	0.007
*R* ^2^	0.888	0.487
PSOM		
*k*_2_ (g/mg·g)	6.62 × 10^−4^	4.99 × 10^−3^
*q_e_* (mg/g)	127.55	126.58
*R* ^2^	0.999	0.999
IPDM (stage 1)		
*k_id_* (g/mg·min^0.5^)	17.41	26.09
*C*	0.2966	4.3046
*R* ^2^	0.999	0.909
IPDM (stage 2)		
*k_id_* (g/mg·min^0.5^)	4.74	1.92
*C*	62.46	106.96
*R* ^2^	0.976	0.972

**Table 4:** Isotherm parameters for dye adsorption onto rice bran/SnO_2_/Fe_3_O_4_.

Dye	RB4				CV			
Temperature (°C)	30	40	50	60	30	40	50	60
Langmuir								
*q_m_* (mg/g)	200.40	204.08	217.39	218.82	138.31	138.50	159.24	139.28
*K_L_* (L/g)	0.027	0.037	0.048	0.054	0.117	0.103	0.567	0.103
*R* ^2^	0.988	0.992	0.997	0.997	0.993	0.992	0.993	0.990
Freundlich								
*K_F_* (mg/g) (L/mg)^1/*n*^	53.48	64.37	74.11	77.43	71.78	73.79	77.80	45.81
*n*	4.74	5.34	5.57	5.67	5.78	5.81	5.65	3.52
*R* ^2^	0.974	0.965	0.998	0.992	0.859	0.917	0.971	0.968

**Table 5:** Comparison of dye adsorption onto different adsorbents.

Adsorbent	Dye	Concentration (mg/L)	pH	Adsorbent Dose (g/L)	T (K)	*q_m_* (mg/g)	Reference
rice husk	CV	50	8	1	303	44.87	[10]
Sawdust	CV	100	-	4	300	37.83	[49]
papaya seed	CV	50	8	12	298	85.99	[50]
tea waste	CV	20	10	1	298	113.64	[51]
rice bran/SnO_2_/Fe_3_O_4_	CV	200	9	1.5	323	159.24	This study
Barley straw	RB4	100	3	2	298	31.5	[52]
Pecan shells	RB4	1000	6.5	10	303	5.0	[53]
peanut hull	RB4	50–350	0.5	1	333	55.55	[54]
rice bran	RB4	200	2	1.5	323	78.0	This study
rice bran/Fe_3_O_4_	RB4	200	2	1.5	303	185.19	Prior study [12]
rice bran/SnO_2_/Fe_3_O_4_	RB4	200	2	1.5	333	218.82	This study

**Table 6:** Analysis of variance (ANOVA) for the empirical model.

Source	RB4 Removal (%)					
	SS	DF	MS	F Value	*p*-Value	
Model	9677.16	9	1075.24	98.84	<0.0001	significant
A	679.99	1	679.99	62.50	<0.0001	significant
B	439.90	1	439.90	40.44	<0.0001	significant
C	7927.37	1	7927.37	728.69	<0.0001	significant
AB	1.35	1	1.35	0.12	0.7317	not significant
AC	125.85	1	125.85	11.57	0.0068	significant
BC	97.51	1	97.51	8.96	0.0135	significant
A^2^	77.40	1	77.40	7.11	0.0236	significant
B^2^	7.831× 10^−9^	1	7.831× 10^−9^	7.198 × 10^−10^	1.0000	not significant
C^2^	293.30	1	293.30	26.96	0.0004	significant
Residual	108.79	10	10.88			
Lack of Fit	83.40	5	16.68	3.28	0.1089	not significant

Note: *R*^2^ = 0.9889, Adj *R*^2^ = 0.9789, Pred *R*^2^ = 0.9311, and adequate precision = 34.747.
